# Supplementary material for: Influence of heat stress on reference genes stability in heart and liver of two chickens genotypes
Source: PLoS One. 2020 Feb 6;15(2):e0228314. doi: 10.1371/journal.pone.0228314 (PMC7004300; doi:10.1371/journal.pone.0228314)
Supplement: Attachment — Submitted filename: Response to Reviewers.docx [file pone.0228314.s002.docx]

Ilhéus, December 18^th^, 2019.

Joerg Heber

Editor-in-Chief

PLOS ONE

We thank the editorial board for your interest in our manuscript entitled “INFLUENCE OF HEAT STRESS ON REFERENCE GENES STABILITY IN HEART AND LIVER OF COMMERCIAL AND BACKYARD CHICKENS”. We are pleased to have the opportunity to return the revised manuscript to Plos One for appreciation. Together with the revised version of the manuscript, we include below a point-by-point reply to the comments of reviewers and details of the modifications made.

We believe that we have now satisfactorily addressed all the issues that were raised by the reviewers.

**Reviewer 1**

**R: Title could be modified to read "Influence of heat stress on reference genes stability in heat and liver of two chicken genotypes”**

Authors: Following your suggestions, we changed the title to "Influence of heat stress on reference genes stability in heart and liver of two chicken genotypes".

**R: Line79-80: Delete**

Authors: We have deleted the sentence and replaced by another sentence emphasizing the importance to perform priori studies to assay the best reference genes for each study, following the suggestions of another reviewer. Please see L79-82.

**R: Line 84 insert "quantitative" between Real-time and polymerase.**

Authors: We have added the information. Please see L86.

**R: Line 86 Replace sentence with "As RT-qPCR is"...**

Authors: We have made the change. Please see L88.

**R: Line 126 Replace "birth" with "hatch"**

Authors: We have made the change. Please see L128.

**R: Line 131 Delete "(wood chips)"**

Authors: We have made the change. Please see L133.

**R: Line 137 should be modified to read "with ad libitum access to water and feed". Delete In a second moment.**

Authors: We have made the changes. Please see L139.

**R: Please ages of the chickens when they were euthanized.**

Authors: All birds in the experiment were euthanized at 30 days of age, as mentioned in the manuscript Material and Methods section. Please see L136-137 in the revised version.

**R:Line 139-141. Please rephrase sentence. Observation cannot prevent death.**

Authors: We rewrote the sentence following your suggestion. Please see L141-142.

**R: How was heart rate measured?**

Authors: There was a mistake in the manuscript writing. The heat stress period was ended when the birds presented accelerated respiratory rate rather than increased heart rate. The information was corrected in the manuscript (see L142-143).

**R:Line 142 Change "slaughtered" to "euthanized"**

Authors: We have made the changes. Please see L144 and L145. In addition, we also have made the change in Abstract section (L58).

**R:Line 147 Change "slaughter" to "euthanization" and "fragments" to "tissues"**

Authors: We have made the changes. Please see L149. In addition, we also have changed "slaughter" to "euthanization" in L137.

**R:Line 167 Delete "in literature"**

Authors: We have made the change. Please see L170-171.

**R:Line 174/200 Change real-time..PCR to RT-qPCR**

Authors: We have made the changes. Please see L178-179 and L204.

**R:Line 184 Change EAU to USA**

Authors: We have made the change. Please see L188.

**Analysis**

**R: The results show a clear genotype by treatment interaction and the results have to be presented as such: Cobb (comfort), Cobb (Heat stress), Peloco (comfort) and Peloco (Heat stress). The authors subsequently make a case for that. Line412-414 "the amount of transcripts may differ in comfort and heat stress, as well as in different genetic groups"**

Authors: Thank you for your remark. Although we understand your point of view, the results were presented in order to enable comparisons between the environments (comfort x stress) and between the genetic groups (Cobb x Peloco). Since different algorithms were used for the analysis, we chose to show the results segregating the tables by tools, so that the results could be clearer. It is worthy to mention that analyses were performed by factors (Cobb 500^®^ and Peloco; comfort and acute heat stress), and subsequently, based on these analyses by factors, an overall ranking was obtained.

**Results**

**R: Line 253-255 Delete or move to discussion. You are expected to present results from the current study. Also, Line 265-266.**

Authors: We have made the changes. Please see L259 and L286.

**Line 264. Insert Table 3 after respectively.**

Authors: We have made the change following your suggestion. Please see L280-285.

**R: It is not clear how Tables 2-5 were generated. They show expression in a tissue of a genotype in two environments. Are the values in the two environments averaged? or one is expressed over the other? Authors should explain what the values represent with respect to the two temperatures.**

Authors: Tables 2-5 were generated based on the results of the BestKeeper algorithm, which performs a descriptive analysis of reference genes expression values and variabilities using Ct (cycle threshold) values. However, this descriptive analysis was performed by genetic group and tissue and using the average values of the two environments, since one of our aims was to compare both groups and tissues.

**Discussion**

**R: Line 406-408 The results in the current study is not in agreement as different tissues were used. The sentence could be restated as "Nascimento et al. [19] also reported that ACTB gene was the least stable in Gallus gallus Pectoralis muscle.**

Authors: We appreciate your suggestion. The change has been made. Please see L415-416.

**R: Line 433 RPL5 second to?**

Authors: In this sense, we realized that the reference gene tested in Nygard et al. (2007) (*RPL4*) was different from our study (*RPL5*), which impairs the comparisons with our study. Therefore, we decided to delete this comparison from the manuscript.

**R: Line 441 EEF1 second most stable compared to?**

Authors: In the study of Kishore et al. (2013), the *EEF1* gene was the seventh most stable in buffalo under heat stress conditions among 11 RG analyzed. The information was corrected and completed in the manuscript (see L447- 448).

**Line 462 Replace "impairing" to "limiting"**

Authors: We have made the change. Please see L468.

**R: Line 474. Provide examples and references.**

Authors: Following your suggestion, new examples and references from recently published articles have been added. See L481-488.

**R: Comfort can be changed to Thermoneutral throughout the text.**

Authors: Dear reviewer, thank you for your suggestion. However, we decided to keep the “Comfort” word throughout the text, since we consider this is a more appropriate word for our study.

**Reviewer 2**

**R:The manuscript by Gromboni et al reports a study to identy reference genes in two tissues for assaying heat stress in chicken. While the study is conducted very well, and reported very clearly, the effort required to identify reference genes - clearly an exercise required for every study - basically argues that this type of approach is required for every study with any variation in breeds, stressors, or tissue sources. This conclusion then greatly limits the utility of this report, except arguing for the need for a priori studies to assay RG at the outset of each study. In this reviewers opinion, these should be at least, among the main conclusions of this report.**

Authors: We agree with the reviewer and it is worthy to mention that there is no a universal RG, and analyzing several factors in different tissues of different organisms that are constantly adapting to changing conditions, different RG expression profiles can be observed. Therefore, the need to perform priori studies to assay the best RG at the outset of each study is emphasized. The suggestion was included in the Conclusion section of the Abstract (see L79-82) and in the main text of the manuscript (see L512-516).

**R: L134: This reviewer has a bit of hesitation accepting that 39.5c is the only metric applied for heat stressed. The temperature is quite a bit off from the higher limits of the chicken thermoneutral zone. While it is reasonable to assume that elevated temperatures cause thermal stress, as far as a phenotypic classification for assessing stability of transcripts, this critiera is insufficient. They mention behavior as a stress marker, but if they had either a serum corticosterone or HSP titer, this categorization would be appropriate. Without this type of secondary confirmation of a heat stress state, we are just looking at expression stability at two differen ambient temperature ranges. Similarly for the ‘comfort’ state. If we are to accept these a phenotypic states, they need to be clearly defined.**

Authors: Dear reviewer, we understand your point of view. However, the results of other study developed by our research group, published in Plos One and of which I am co-author (Cedraz et al., 2017), showed that the temperature of 39.5°C was sufficient to induce heat stress in the birds. It is important to mention that in Cedraz et al. (2017), the same genetic groups and environmental conditions of the present study were used and the expressions of heat stress markers (HSF1, HSF3, HSP70 and HSP90) were evaluated. The reference of the mentioned study follows in sequence.

Reference

CEDRAZ, H. et al. Heat stress induces expression of HSP genes in genetically divergent chickens. Plos One v.12 n.10, 2017.

**R: L154: What was the RNA quality? What criteria were applied to determine if quality was sufficient for RT-qPCR?**

Authors: Great care is required when working with RNA, since it is extremely susceptible to degradation due to the action of RNases. Therefore, its quality should be checked before RT-qPCR is performed. In the present study, RNA extraction was firstly performed in both tissues, but there was a variation in concentration between tissues. In heart tissue, the concentration was on average 800 ng/µl, while in liver the average concentration was 3000 ng/µl. To standardize the amount of RNA for the study, following the reagent protocol, all samples were diluted to a concentration of 500 ng/µl for subsequently cDNA synthesis. The average absorbance ratios at 260 and 280 nanometers were excellent, in general higher than 1.8. In addition, after extraction, the rRNA bands were checked after the samples stained with ethidium bromide were submitted to 1% agarose gel electrophoresis and visualized in ultraviolet light, confirming the samples integrity. In this way, the RNA concentration, integrity and quality were checked and established to be sufficient for RT-qPCR. We have added information regarding the criteria used to determine the RNA quality in the text (see L154-158).

**R:** **L167 Why were these reference genes selected? Please add additional details.**

Authors: These reference genes were selected based on their biological importance. They participate in primary metabolism and maintenance of cell structure. In addition, our research group had already performed other study using these genes primers, which was published in Plos One (Cedraz et al., 2017). We appreciate your remark and the information has been added to the manuscript (See L170-171).

Reference

CEDRAZ, H. et al. Heat stress induces expression of HSP genes in genetically divergent chickens. Plos One v.12 n.10, 2017.

**R: L184: Please change ‘EUA’ to ‘USA’**

Authors: We have made the change. Please see L188.

**R: Table 1: Please provide either the ENSEMBL gene id or transcript id. The IDs reported in this table are not correct ensembl ids.**

Authors: Thank you for your observation. Indeed, the *ACTB* gene ID was incorrectly typed. All genes IDs were checked and then deposited in NCBI and ENSEMBL. See Table 1 in the revised version of the manuscript.

**R: L202: Non template controls were not used. This is an important experimental design oversight.**

Authors: Dear reviewer, we understand your point of view. Nevertheless, we considered the Cobb 500^®^ commercial line under thermal comfort condition as the control group.

**R: With so many markers used, please provide additional details on the plate set-up of the reactions. Were comfort time samples included on all plates?**

Authors: We appreciate your suggestion. Additional information on the plate set-up of the reactions has been added in the manuscript (See L176-179). Regarding the comfort time samples, all of them were included in all plates, in duplicates, for all markers.

**R: L497: While the methodology and the analysis show that the RPL5, EEF1, and LDHA genes are the best reference genes for assessing heat stress in these tissues, the main takeaway for this reviewer hava been that a) reference genes may have to be evaluated for every study based on breed, stressor, and tissue sampled, and b) the use of the RankAggreg tool is required to come to this decision. These efforts suggest that the amount of work required to identify a correct RG is extremely high, in proportion to the value of the information revealed. Overall, to this reviewer, this paper argues against the use of qPCR methods for assaying phenotypic states in chicken tissues.**

Authors: After obtaining the Ct (Cycle threshold) values of all samples (breed, stressor and tissue), the data were separately evaluated by factor (Cobb 500^®^ and Peloco; comfort and acute heat stress), and, posteriorly, the RankAggreg tool was used to decide that the *RPL5, EEF1* and *LDHA* genes were the most stable in all situations. Our idea was not to argue against the use of qPCR methods. One of our goals was to highlight the importance of normalization to validate the gene expression results, seeking to ensure that any variation in the genetic material is related to the same extent between the research object and the control. In addition, some authors report that no gene is constitutively expressed in all tissue types and under all experimental conditions, requiring a species-specific determination, since a reference gene may be suitable for one specie and not for another (BRUNNER; YAKOVLEV; STRAUSS, 2004). Therefore, there need to evaluate the stability of reference genes expression prior to their use in gene expression analysis is emphasized.

References

Bruner AM, Yakovlev IA, Strauss SH. Validating internal controls for quantitative plant gene expression studies. BMC Plant Biology. 2004; 4 (1):14.

**R: L506: misspelled.**

Authors: Thank you for your observation. The word was corrected (See L519).

Sincerely yours,

Juliana Gracielle Gonzaga Gromboni

e-mail: jugracielle@hotmail.com
